# Renal size and cardiovascular risk in prepubertal children

**DOI:** 10.1038/s41598-019-41757-2

**Published:** 2019-03-27

**Authors:** Esther Lizarraga-Mollinedo, Jose-Maria Martínez-Calcerrada, Cristina Padrós-Fornieles, Berta Mas-Pares, Silvia Xargay-Torrent, Elena Riera-Pérez, Anna Prats-Puig, Gemma Carreras-Badosa, Francis de Zegher, Lourdes Ibáñez, Judit Bassols, Abel López-Bermejo

**Affiliations:** 1 Pediatric Endocrinology Group, [Girona Biomedical Research Institute] IDIBGI, Salt, 17190 Spain; 2 Maternal-Fetal Metabolic Group, [Girona Biomedical Research Institute] IDIBGI, Salt, 17190 Spain; 3 Pediatrics, Dr. Trueta University Hospital, Girona, 17007 Spain; 4 Pediatrics, Salut Empordà Foundation, Figueres, 17600 Spain; 5 Department of Physical Therapy, EUSES University School, Salt, 17190 Spain; 6 Department of Development & Regeneration, University of Leuven, Leuven, 3000 Belgium; 7 Endocrinology, Pediatric Research Institute Sant Joan de Déu, 08950 Esplugues, Spain; 8 [Spanish Biomedical Research Centre in Diabetes and Associated Metabolic Disorders] CIBERDEM, ISCIII, Madrid, 28029 Spain

## Abstract

Renal size is an important parameter for the evaluation and diagnosis of kidney disease and has been associated with several cardiovascular risk factors in patients with kidney failure. These results are however discordant and studies in healthy children are lacking. We aimed to study the association between renal size (length and volume) and cardiovascular risk parameters in healthy children. Clinical, analytical and ultrasound parameters [renal length, renal volume, perirenal fat and carotid intima-media thickness (cIMT)] were determined in 515 healthy prepubertal children (176 lean, 208 overweight and 131 obese). Renal length and volume associated significantly and positively with several anthropometric and cardiovascular risk parameters including cIMT and systolic blood pressure (SBP) (all p < 0.001). Renal length and volume associated with cIMT and SBP in all study subgroups, but these associations were predominant in obese children, in whom these associations were independent after adjusting for age, gender and BSA (all p < 0.05). In multivariate analyses in the study subjects as a whole, renal length was an independent predictor of cIMT (β = 0.310, p < 0.0001) and SBP (β = 0.116, p = 0.03). Renal size associates with cIMT and SBP, independent of other well-established cardiovascular risk factors, and may represent helpful parameters for the early assessment of cardiovascular risk in children.

## Introduction

The kidney plays a central role in the regulation of electrolyte homeostasis and blood pressure and its dysregulation has been associated with several cardiovascular diseases (CVD)^1–3^.

Arterial vascular disease, characterized by increased systolic blood pressure and atherosclerosis, are the primary types of CVD present in patients with kidney failure^4^. Atherosclerosis is an intimal disease produced by the presence of plaques that cause occlusive lesions^5^. A surrogate marker of atherosclerosis is the intima-media thickness of the carotid wall (cIMT)^6^. cIMT is easily detectable by ultrasound and is also regarded as an indicator of preclinical atherosclerosis in children^7^.

In fact, renal size may be a marker for the loss of kidney mass and function^8,9^, as several authors have described associations between renal volume, nephron mass and kidney disease^10–12^. Besides renal volume, renal length has also been associated with nephron mass and renal function (estimated by GFR)^13–15^. In children, the studies are scarce but indicate that renal size positively correlates with its functionality^16^.

Several studies have examined the relationship between blood pressure and kidney measurements in different groups of patients^17–20^; however, there is a great discrepancy in the results derived from these studies and no investigations have been performed in healthy children. cIMT has been associated with renal function in adults^21–24^ and perirenal fat in children^25^; however, studies about the association between cIMT and renal size are lacking.

Given that there is an accumulating body of evidence suggesting an association between kidney measurements and CVD, and that cardiovascular risk factors may be present in early life, we aimed to study the association between renal size (length and volume) and cardiovascular risk parameters (cIMT and SBP) in children in order to identify novel markers that can be easily assessed and used in the early prevention of CVD.

## Results

Clinical, laboratory and ultrasonography assessments are shown in the studied subjects as a whole and in subgroups thereof according to BMI (lean, overweight and obese) (Table 1). Renal parameters (length, volume and perirenal fat) were higher in overweight and obese, compared to lean children (all p < 0.0001) and in obese, compared to overweight children (all p < 0.0001).

**Table 1:** Clinical, laboratory and ultrasonography assessments in the studied subjects as a whole and in subgroups thereof according to BMI (lean, overweight and obese).

	All subjects (n = 515)	Lean (BMI-SDS <1) (n = 176)	Overweight (1< BMI-SDS <2) (n = 208)	Obese (BMI-SDS >2) (n = 131)
**Clinical assessments**				
Age (year)	8.7 ± 0.1	8.1 ± 0.2	8.9 ± 0.1**	9.3 ± 0.2**
Gender (%F)	45.0	46.0	45.7	42.7
Birth weight (kg)	3.1 ± 0.1	2.9 ± 0.1	3.1 ± 0.1*	3.3 ± 0.1**
Birth weight-SDS	0.09 ± 0.07	−0.41 ± 0.1	0.27 ± 0.1**	0.54 ± 0.2**
Weight (kg)	39.7 ± 0.7	26.0 ± 0.6	41.8 ± 0.8**	54.9 ± 1.3**^ππ^
Weight-SDS	0.95 ± 0.07	−0.62 ± 0.05	1.16 ± 0.05**	2.74 ± 0.08**^ππ^
Height (cm)	135.6 ± 0.6	128.3 ± 1.1	137.7 ± 0.9**	142.1 ± 1.1**^ππ^
Height-SDS	0.53 ± 0.05	−0.03 ± 0.09	0.68 ± 0.08**	1.05 ± 0.1**^π^
BMI (kg/m^2^)	20.75 ± 0.21	15.4 ± 0.1	21.5 ± 0.2^*^*	26.7 ± 0.3**^ππ^
BMI-SDS	0.85 ± 0.06	−0.8 ± 0.03	1.1 ± 0.04**	2.7 ± 0.06**^ππ^
BSA (m^2^)	1.2 ± 0.01	0.9 ± 0.01	1.3 ± 0.02**	1.5 ± 0.02**^ππ^
Fat mass (Kg)	9.8 ± 0.4	4.7 ± 0.3	10.9 ± 0.6**	14.9 ± 1.1**^π^
Waist (cm)	68.2 ± 0.7	54.4 ± 0.4	71.2 ± 0.7**	82.2 ± 1.2**^ππ^
SPB (mmHg)	108.5 ± 0.5	103.7 ± 0.6	109.7 ± 0.7**	113.1 ± 0.9**^ππ^
DBP (mmHg)	62.4 ± 0.4	60.1 ± 0.5	61.9 ± 0.5*	66.5 ± 0.8**^ππ^
**Laboratory assessments**				
Insulin (mIU/L)	6.9 ± 0.3	3.2 ± 0.2	6.9 ± 0.4**	11.5 ± 0.7**^ππ^
HOMA-IR	1.5 ± 0.1	0.7 ± 0.05	1.5 ± 0.08**	2.5 ± 0.2**^ππ^
Triacylglycerol (mg/dL)	66.2 ± 1.6	48.9 ± 1.4	68.4 ± 2.6**	85.2 ± 3.7**^ππ^
HDL-cholesterol (mg/dL)	42.5 ± 0.3	40.7 ± 0.6	43.3 ± 0.5*	43.7 ± 0.6*
Urea (mg/dL)	30 ± 0.3	30.9 ± 0.5	29.7 ± 0.5	29.4 ± 0.6*
eGFR (mL/min per 1.73 m^2^)	150.2 ± 1	148.7 ± 1.7	149.7 ± 1.6	152.9 ± 2.1
ALT (U/L)	19.4 ± 0.4	16.1 ± 0.3	19.2 ± 0.5*	24.3 ± 1.1**^ππ^
GGT (U/L)	13.8 ± 0.2	11.5 ± 0.2	13.8 ± 0.3**	16.9 ± 0.5**^ππ^
**Ultrasonography assessments**				
Renal length (cm)	8.9 ± 0.05	8.2 ± 0.07	9.0 ± 0.06**	9.5 ± 0.09**^ππ^
Renal volume (cm^3^)	81.2 ± 1.2	67.2 ± 1.5	83.1 ± 1.7**	97.2 ± 2.5**^ππ^
Perirenal fat (cm)	0.20 ± 0.002	0.18 ± 0.004	0.20 ± 0.004*	0.23 ± 0.005**^ππ^
cMT (cm)	0.040 ± 0.000	0.038 ± 0.000	0.040 ± 0.000*	0.043 ± 0.000**^ππ^

Data are shown as mean ± SEM. BMI, body mass index; SDS, standard deviation score; BSA, body surface area; SBP, systolic blood pressure; DBP, diastolic blood pressure; eGFR, estimated glomerular filtration rate; ALT: alanine-aminotransferase; GGT: gamma-glutamyl transferase; cIMT, carotid intima-media thickness. **p < 0.0001 vs Lean, *p < 0.05 vs Lean, ^ππ^p < 0.0001 vs Overweight, ^π^p < 0.05 vs Overweight.

In all the studied subjects, renal length and renal volume associated significantly and positively with several anthropometric and cardiovascular risk parameters including weight-SDS, height-SDS, BMI-SDS, BSA, waist, fat mass, SBP, DBP, insulin, HOMA-IR, TG, creatinine and cIMT and negatively with HDL-cholesterol (all p < 0.001) (Table 2).

**Table 2:** Correlation coefficients for renal length and renal volume with clinical, laboratory and ultrasonography assessments in the study subjects.

	Renal length		Renal volume	
	r	P	r	P
**Clinical assessments**				
Age (year)	0.619	<0.0001	0.641	<0.0001
Birth Weight-SDS	0.235	<0.0001	0.216	<0.0001
Birth Height-SDS	0.262	<0.0001	0.229	<0.0001
Weight-SDS	0.520	<0.0001	0.503	<0.0001
Height-SDS	0.370	<0.0001	0.337	<0.0001
BMI-SDS	0.483	<0.0001	0.470	<0.0001
BSA (m^2^)	0.740	<0.0001	0.762	<0.0001
Waist (cm)	0.615	<0.0001	0.604	<0.0001
Fat Mass (Kg)	0.426	<0.0001	0.471	<0.0001
SPB (mmHg)	0.458	<0.0001	0.450	<0.0001
DBP (mmHg)	0.217	<0.0001	0.213	<0.0001
**Laboratory assessments**				
Insulin (mIU/L)	0.410	<0.0001	0.451	<0.0001
HOMA-IR	0.409	<0.0001	0.447	<0.0001
Triacylglycerol (mg/dL)	0.233	<0.0001	0.243	<0.0001
HDL-cholesterol (mg/dL)	−0.297	<0.0001	−0.263	<0.0001
Creatinine (μmol/L)	0.314	<0.0001	0.309	<0.0001
Urea (mg/dL)	−0.088	0.04	−0.051	Ns
eGFR (mL/min)	0.123	0.005	0.123	0.005
ALT (U/L)	0.285	<0.0001	0.321	<0.0001
GGT (U/L)	0.361	<0.0001	0.390	<0.0001
**Ultrasonography assessments**				
Renal length (cm)	—	—	0.758	<0.0001
Renal volume (cm^3^)	0.758	<0.0001	—	—
Perirenal fat (cm)	0.422	<0.0001	0.397	<0.0001
cIMT (cm)	0.408	<0.0001	0.311	<0.0001

p and r values are from Pearson correlation analyses. BMI, body mass index; SDS, standard deviation score; BSA, body surface area; SBP, systolic blood pressure; DBP, diastolic blood pressure; eGFR, estimated glomerular filtration rate; ALT: alanine-aminotransferase; GGT: gamma-glutamyl transferase; cIMT, carotid intima-media thickness.

**Figure 1:**
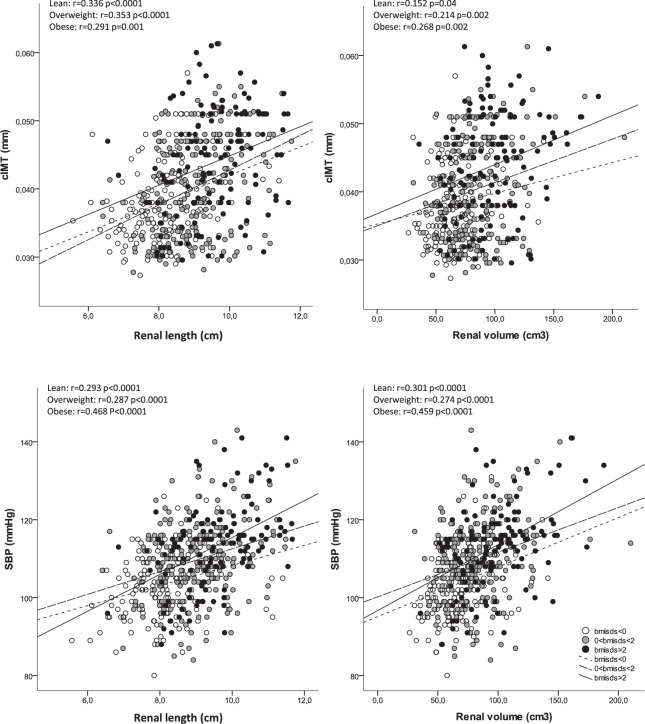
Correlations between renal length and renal volume with cIMT and SBP according to BMI categories. White dots and dotted lines indicate lean children (BMI-SDS < 0), grey dots and dashed line indicate overweight children (0 < BMI-SDS < 2) and black dots and continuous line indicate obese children (BMI-SDS ≥ 2).

The associations of renal length and renal volume with cIMT and SBP were significant in all BMI subgroups (all between p < 0.05 and p < 0.0001), although they were predominant (higher correlation coefficients) in obese children (Fig. 1 and Table 3). A number of these associations were maintained after adjusting for age, gender and BSA (Table 4). Renal length associated with cIMT in all studied subgroups and with SBP in obese children, while renal volume associate with cIMT and SBP in obese children (all p < 0.05). We also evaluated the interaction effect of obesity in theses associations and the results showed a significant interaction of obesity in the association between renal length and SBP (p = 0.03).

**Table 3:** Correlation coefficients for cardiovascular risk factors (cIMT and SBP) with renal parameters in the study subjects as a whole and in subgroups thereof according to BMI (lean, overweight and obese).

	All children (n = 515)		Lean (BMI-SDS <1) (n = 176)		Overweight (1< BMI-SDS <2) (n = 208)		Obese (BMI-SDS >2) (n = 131)	
cIMT	r	p	r	p	r	p	r	p
Renal length	0.408	<0.0001	0.336	<0.0001	0.353	0.001	0.291	0.001
Renal volume	0.311	<0.0001	0.152	0.04	0.214	0.002	0.268	0.002
**SBP**	**r**	**p**	**r**	**p**	**r**	**p**	**r**	**p**
Renal length	0.458	<0.0001	0.293	<0.0001	0.287	<0.0001	0.468	<0.0001
Renal volume	0.450	<0.0001	0.301	<0.0001	0.274	<0.0001	0.459	<0.0001

p and r values are from Pearson correlation analyses. cIMT: carotid intima-media thickness, SBP: systolic blood pressure.

**Table 4:** Multivariate linear regression analysis for cardiovascular risk factors (cIMT and SBP) and renal parameters adjusted for age, gender and BSA in the study subjects as a whole and in subgroups thereof according to BMI (lean, overweight and obese).

	All children (n = 515)		Lean (BMI-SDS <1) (n = 176)		Overweight (1< BMI-SDS <2) (n = 208)		Obese (BMI-SDS >2) (n = 131)	
cIMT	B	p	B	p	B	p	B	p
Renal length	0.376	<0.0001	0.347	<0.0001	0.410	<0.0001	0.309	0.008
Renal volume	0.154	0.01	—	—	—	—	0.269	0.03
**SBP**								
Renal length	0.120	0.02	—	—	—	—	0.277	0.008
Renal volume	—	—	—	—	—	—	0.235	0.04

cIMT: carotid intima-media thickness, SBP: systolic blood pressure, BSA: body surface area.

We were also interested to know if both renal length and perirenal fat were able to independently predict these cardiovascular risk parameters. To this aim, we performed additional multiple regression analyses of cIMT and SBP as dependent variables in the whole group of studied children. In these models, we computed both renal length and perirenal fat as independent variables, together with age, gender, BSA, and serum lipids. The regression model of cIMT showed that renal length (β = 0.310, p < 0.0001) and perirenal fat (β = 0.233, p < 0.0001) were independent predictors of cIMT and explained 21.1% of the variance in cIMT. The regression model of SBP showed that renal length (β = 0.116, p = 0.03) and BSA (β = 0.463, p < 0.0001) were independent predictors of SBP and explained 30.7% of the variance in SBP (Table 5).

**Table 5:** Multivariate linear regression analyses for cardiovascular risk factors (cIMT and SBP) and renal parameters after further adjusting for perirenal fat and serum lipids in the studied subjects as a whole (n = 515).

	Beta	Sig
**cIMT**		
Renal length	0.310	<0.0001
Perirenal fat	0.233	<0.0001
R^2^	0.211	
**SBP**		
Renal length	0.116	0.03
BSA	0.463	<0.0001
R^2^	0.307	

cIMT, carotid intima-media thickness; SBP, systolic blood pressure;

Non predictive variables: age, gender, renal volume and lipids.

## Discussion

Our study showed that renal size associated with cIMT and SBP, independently of other well-established risk factors such as age, gender, BSA and serum lipids in healthy prepubertal children.

The size of the kidney varies and is mainly affected by age, gender, height and BMI^26^. Kidney size provides a rough indication of renal function and seems to be related to a number of risk factors for cardiovascular disease; however, the studies reporting relationships between renal size and blood pressure have shown controversial findings. For instance, renal volume adjusted for sex, age and BSA was inversely correlated with SBP in a study of Australian aboriginal children and adults (n = 668)^19^. These results could be attributable to the rather low birth weight of the studied subjects, a common characteristic (up to 60%) among aboriginal people. It is known that low nephron numbers and higher blood pressure could be caused by fetal adaptation to intrauterine growth-restriction^27^. However, Gurusinghe *et al*.^18^ showed that total kidney volume was positively associated with SBP indices in children below 21 years (n = 84) diagnosed with primary hypertension, contrary to which they were expecting since hypertensive patients are found to have fewer glomeruli than their normotensive counterparts^28^. They claimed that low birth weight may have a greater impact on BP with increasing age^29^. Yet, other studies disclosed that there is no significant correlation between blood pressure and renal size in healthy adults (n = 185), after correcting for age, sex and BMI^30^. Similarly, renal volume was related to renal function but not with blood pressure in a population-based cohort study of children (n = 6397)^31^. In a study of hypertensive and control adult patients (n = 195)^32^, the authors found a correlation between BMI, birth weight, renal volume and blood pressure only in the hypertensive group.

Our findings in healthy prepubertal children showed that renal length and volume associate with SBP mainly in obese children. In fact, it is known that overweight and obese subjects have greater carotid diameters and measures of adiposity and higher SBP^33^. These associations were not explained by body surface, as they were independent of BSA in our study population.

Interestingly, similar associations were observed between renal length and volume and cIMT. As far as we are aware, this is the first description of an independent association between renal size and cIMT in healthy children. Indeed, previous works have focused on the association between several cardiovascular risk markers and impairment of renal function in adult patients. In hypertensive patients, cIMT correlated with increased concentration of cystatin C (n = 87)^34^ and, in another study, cIMT correlated with low eGRF (n = 1351)^23^. Others showed that atherosclerosis was a predictor of renal size and function in patients with atherosclerotic vascular disease (n = 1056)^22^. A lack of independent relationship between renal function and atherosclerosis in subjects with normal renal function was also described^23,24^. Similar results were observed in our cohort of healthy prepubertal children. eGRF correlated with cIMT; however, the associations were not maintained in multivariate analysis including BSA and other confounding variables.

Our multivariate analyses showed that renal length together with perirenal fat were independent predictors of cIMT. Importantly, we have previously demonstrated a strong association between perirenal fat and cIMT, considering perirenal fat as a cardiovascular risk enhancer^25^. We suggested that the harmful impact of perirenal fat could be related to the local synthesis of adipocytokines that may impact on renal function. Renal size has also been previously proposed to play a role in estimating renal function in healthy children^16^. Hence, the present results pointed that perirenal fat and renal size could both modulate vascular function and thus be associated with cardiovascular risk. In these sense, there is a study in Ossabaw pigs with metabolic syndrome that shows that greater renal size and higher glomerular filtration rate associated with microvascular proliferation and proximal tubular vacuolization, as well as significant intrarrenal and perirenal adiposity^35^.

We have used renal length and volume to assess renal size. Both measures correlated with cIMT and SBP; however, in multivariate analysis, renal length, but no renal volume, was an independent predictor of cIMT and SBP. This discrepancy could be explained because of the better repeatability and accuracy of renal length measurements compared to renal volume. Several authors suggest that renal length may be a better marker than renal volume because is easily reproduced and less technically demanding than renal volume, which requires several measurements in different planes and the application of a formula^19,36^.

We recognize a number of limitations in our study. In particular, the study has a cross sectional design, therefore it is not possible to explore a causative relationship between renal size and cardiovascular measures. However, this study seeks an interesting age group since atherosclerosis begins in childhood^7^. Moreover, children are not subjected to potential confounding conditions such as adult-onset metabolic complications. Our findings are additionally limited to a pediatric population of 515 healthy patients, indicating that future investigations are needed to confirm the link between renal length and cardiovascular risk. We have observed significant independent associations between renal size (length and volume) and SBP but not with DBP; however, SBP is considered to be a better parameter associated with CV risk^37,38^. Regarding the methodology, we have not measured intrarenal resistive index and even though both kidneys were measured, we only show data for the right one, as the measurements of the left kidney were less reproducible in our study. Blood pressure was only measured on the right arm; however, all subjects had normal femoral pulse suggesting that none had aortic coarctation. Our study may have not excluded subjects with spurious hypertension, but we could not determine central SBP because it is an invasive measurement. cIMT was only measured on the right artery; however, we have shown consistent results regarding the association of cIMT with CV risk in a number of manuscripts^39,40^. No external validations for the ultrasound measurements were performed; however, cIMT and renal size have been shown to be easily and reproducibly measured by ultrasound in numerous studies^41,42^.

In conclusion, our findings showed that renal size associated with cIMT and SBP, independently of other well-established risk factors. Renal length and perirenal fat were independent predictors of cIMT, while renal length and BSA were independent predictors of SBP. We propose that these renal parameters may play a role in the regulation of vascular function and may represent helpful parameters for the early prevention of cardiovascular risk in children.

## Methods

### Subjects and Ethics

The study population consisted of 515 asymptomatic Caucasian children (283 boys and 232 girls; mean age 8.7 ± 0.1 years); 176 lean (BMI-SDS < 1); 208 overweight (1 ≤ BMI-SDS < 2) and 131 obese (BMI-SDS ≥ 2). Subjects were enrolled in a study of obesity and cardiovascular risk factors in prepubertal childhood and consecutively recruited among those seen in a primary care setting in Girona, a region in Northeastern Spain.

The inclusion criteria were 1) age between 6 and 10 years and 2) prepubertal status (Tanner stage I). Children with major congenital anomalies; abnormal liver, kidney or thyroid functions; evidence of chronic illness or prolonged use of medication; acute illness or use of medication in the month preceding potential enrolment were excluded from the study.

The study protocol was approved by the Institutional Review Board of Dr Josep Trueta Hospital. All research was performed in accordance with relevant guidelines and regulations. Informed written consent was obtained from all the parents.

### Clinical assessments

Clinical examination was performed in the morning, in the fasting state, and it was followed by venous blood sampling. Weight and height were measured with a calibrated scale and a Harpenden stadiometer, respectively. Body mass index (BMI) was calculated as weight (in kg) divided by the square of height (in meters). Age-adjusted and sex-adjusted standard deviation scores (SDS) for BMI were calculated using regional normative data^43^. Body surface area (BSA) was calculated as the square root of height (in cm) * weight (in kg)/3600. Waist circumference was measured in the supine position at the umbilical level. Body composition was assessed by bioelectric impedance (Hydra Bioimpedance Analyzer 4200, Xitron Technologies, San Diego, CA). Fat mass (FM) percentage was calculated using the body weight and lean mass parameters [FM = (body weight − lean mass)/*100]. Systolic (SBP) and diastolic (DBP) blood pressure was measured on the right arm after a 10-min rest in the supine position by means of an electronic sphygmomanometer (Dinamap Pro 100, GE Healthcare, Chalfont St. Giles, United Kingdom). It was determined twice and an additional measurement was taken in case there was no agreement between the initial two measurements. Data are presented as the average of two concordant measurements. Information about birth parameters was abstracted from standardized medical records.

### Laboratory assessments

All serum samples were obtained between 8:00 and 9:00 AM under fasting conditions. Fasting serum glucose, immunoreactive insulin, serum alanine-aminotransferase (ALT), gamma-glutamyl transferase (GGT), creatinine, urea and lipids (total cholesterol, triglycerides and HDL-cholesterol) were routinely assessed in the clinical laboratory of the Hospital Dr. Josep Trueta (Architect system, Abbott Diagnostics Europe, Milan, Italy). Insulin resistance was calculated using the homeostasis model assessment of insulin resistance (HOMA-IR = [fasting insulin in mUl^−1^] * [fasting glucose in mM]/22.5). Glomerular filtration rate (GFR) was estimated by the Haycock-Schwartz formula as follows: *K* × height (in cm)/creatinine (in umol/L), with a *K* value of 46 for all ages^44^.

### Ultrasound assessments

Right kidney size (length, depth and width), perirenal fat and cIMT were measured by high-resolution ultrasonography (MyLabTM25, Esaote, Firenze, Italy). Averages of three measurements for each parameter were used in the study. Renal size was measured in both kidneys with the subjects placed in left lateral supine position. Given that the right kidney was more easily assessed, and so these measurements were more reproducible, we only show data of the right one. During ultrasound measurements, participants with abnormal morphometry in any of the kidneys, suggesting renal scar, reflux nephropathy, and those with asymmetric kidneys, were further excluded from the study. Renal volume was calculated using the following formula [length * depth * width * 0.523]^45^. cIMT was measured on the right side at the level of the distal common carotid artery, one cm away from its bifurcation. Diastolic images were obtained using a linear 7.5–12 MHz transducer. Perirenal fat images were obtained using a convex 3–3.5 MHz transducer and fat thickness was measured as the distance from the inner side of the abdominal musculature to the surface of the right kidney. All measurements were performed by the same observer who was unaware of the clinical and laboratory characteristics of the subjects. Intra-subject coefficient of variation for ultrasound measurements was less that 6%. None of the children in our study showed ultrasonographic signs of atherosclerotic plaques.

### Statistics

Statistical analyses were performed using SPSS version 22.0 (SPSS Inc., Chicago, IL). Results are expressed as mean ± standard error of the mean (SEM). Logarithmic transformation was used to obtain normally distributed values for continuous variables. Differences across obesity groups were examined by ANOVA (continuous data). The relation between variables was analyzed by Pearson bivariate correlations followed by multivariate linear regression analyses. The enter method was used for computing the independent variables. Significance level was set at p < 0.05.

## References

[1] Go AS, Chertow GM, Fan D, McCulloch CE, Hsu CY (2004). Chronic kidney disease and the risks of death, cardiovascular events, and hospitalization. The New England journal of medicine.

[2] Coresh J, Astor B, Sarnak MJ (2004). Evidence for increased cardiovascular disease risk in patients with chronic kidney disease. Current opinion in nephrology and hypertension.

[3] Sarnak MJ (2003). Kidney disease as a risk factor for development of cardiovascular disease: a statement from the American Heart Association Councils on Kidney in Cardiovascular Disease, High Blood Pressure Research, Clinical Cardiology, and Epidemiology and Prevention. Circulation.

[4] London GM, Marchais SJ, Guerin AP, Metivier F, Adda H (2002). Arterial structure and function in end-stage renal disease. Nephrology, dialysis, transplantation: official publication of the European Dialysis and Transplant Association - European Renal Association.

[5] Jungers P (1997). Incidence and risk factors of atherosclerotic cardiovascular accidents in predialysis chronic renal failure patients: a prospective study. Nephrology, dialysis, transplantation: official publication of the European Dialysis and Transplant Association - European Renal Association.

[6] de Groot E (2004). Measurement of arterial wall thickness as a surrogate marker for atherosclerosis. Circulation.

[7] Li S (2003). Childhood cardiovascular risk factors and carotid vascular changes in adulthood: the Bogalusa Heart Study. Jama.

[8] Shcherbak, A. Angiographic criteria in the determination of indications for organ-preserving surgery in renal artery occlusion. *Klinicheskaia khirurgiia*. 15–17 (1989) 2632939

[9] Guzman RP, Zierler RE, Isaacson JA, Bergelin RO, Strandness DE (1994). Renal atrophy and arterial stenosis. A prospective study with duplex ultrasound. Hypertension.

[10] Hoy, W. E. *et al*. A stereological study of glomerular number and volume: preliminary findings in a multiracial study of kidneys at autopsy. *Kidney international Supplement*, S31–37 (2003). 10.1046/j.1523-1755.63.s83.8.x 12864872

[11] Hoy WE (2008). Nephron number, glomerular volume, renal disease and hypertension. Current opinion in nephrology and hypertension.

[12] Hoy WE (2011). Distribution of volumes of individual glomeruli in kidneys at autopsy: association with physical and clinical characteristics and with ethnic group. American journal of nephrology.

[13] Paleologo G (2007). Kidney dimensions at sonography are correlated with glomerular filtration rate in renal transplant recipients and in kidney donors. Transplantation proceedings.

[14] Akpinar IN (2003). Sonographic measurement of kidney size in geriatric patients. Journal of clinical ultrasound: JCU.

[15] Kariyanna SS, Light RP, Agarwal R (2010). A longitudinal study of kidney structure and function in adults. Nephrology, dialysis, transplantation: official publication of the European Dialysis and Transplant Association - European Renal Association.

[16] Adibi A, Adibi I, Khosravi P (2007). Do kidney sizes in ultrasonography correlate to glomerular filtration rate in healthy children?. Australasian radiology.

[17] Paivansalo MJ (1998). Effect of hypertension, diabetes and other cardiovascular risk factors on kidney size in middle-aged adults. Clinical nephrology.

[18] Gurusinghe S (2017). Kidney volume and ambulatory blood pressure in children. Journal of clinical hypertension.

[19] Singh GR, Hoy WE (2004). Kidney volume, blood pressure, and albuminuria: findings in an Australian aboriginal community. American journal of kidney diseases: the official journal of the National Kidney Foundation.

[20] Zumrutdal AO, Turan C, Cetin F, Adanali S (2002). Relationship between renal size and hypertension in patients with chronic renal failure. Nephron.

[21] Bobbert T (2010). Relation between physiological variation of renal function and carotid intima media thickness in non-diabetic individuals. Journal of atherosclerosis and thrombosis.

[22] Bax L (2003). Influence of atherosclerosis on age-related changes in renal size and function. European journal of clinical investigation.

[23] Ishizaka N (2007). Association between chronic kidney disease and carotid intima-media thickening in individuals with hypertension and impaired glucose metabolism. Hypertension research: official journal of the Japanese Society of Hypertension.

[24] Han L, Bai X, Lin H, Sun X, Chen XM (2010). Lack of independent relationship between age-related kidney function decline and carotid intima-media thickness in a healthy Chinese population. Nephrology, dialysis, transplantation: official publication of the European Dialysis and Transplant Association - European Renal Association.

[25] Bassols, J. *et al*. Perirenal fat is related to carotid intima-media thickness in children. *International journal of obesity* **42**, 641–647 (2018). 10.1038/ijo.2017.236 29064476

[26] Oswald J (2004). Age and lean body weight related growth curves of kidneys using real-time 3-dimensional ultrasound in pediatric urology. The Journal of urology.

[27] Spencer J, Wang Z, Hoy W (2001). Low birth weight and reduced renal volume in Aboriginal children. American journal of kidney diseases: the official journal of the National Kidney Foundation.

[28] Keller G, Zimmer G, Mall G, Ritz E, Amann K (2003). Nephron number in patients with primary hypertension. The New England journal of medicine.

[29] Moore VM, Cockington RA, Ryan P, Robinson JS (1999). The relationship between birth weight and blood pressure amplifies from childhood to adulthood. Journal of hypertension.

[30] Raman GV, Clark A, Campbell S, Watkins L, Osmond C (1998). Is blood pressure related to kidney size and shape?. Nephrology, dialysis, transplantation: official publication of the European Dialysis and Transplant Association - European Renal Association.

[31] Bakker H (2014). Kidney size and function in a multi-ethnic population-based cohort of school-age children. Pediatric nephrology.

[32] Laganovic M (2009). Kidney volume and albuminuria as markers of birth weight-blood pressure relationship in essential hypertension. Kidney & blood pressure research.

[33] Scuteri A (2012). Associations of large artery structure and function with adiposity: effects of age, gender, and hypertension. The SardiNIA Study. Atherosclerosis.

[34] Skalska A, Klimek E, Wizner B, Gasowski J, Grodzicki T (2007). Kidney function and thickness of carotid intima-media complex in patients with treated arterial hypertension. Blood pressure.

[35] Li Z (2011). Increased glomerular filtration rate in early metabolic syndrome is associated with renal adiposity and microvascular proliferation. American journal of physiology Renal physiology.

[36] Bakker J (1999). Renal volume measurements: accuracy and repeatability of US compared with that of MR imaging. Radiology.

[37] Black HR (2004). The paradigm has shifted to systolic blood pressure. Journal of human hypertension.

[38] Stabouli S (2015). Arterial stiffness and SBP variability in children and adolescents. Journal of hypertension.

[39] Osiniri I (2012). Carotid intima-media thickness at 7 years of age: relationship to C-reactive protein rather than adiposity. The Journal of pediatrics.

[40] Bassols J (2018). Perirenal fat is related to carotid intima-media thickness in children. Int J Obes (Lond).

[41] Dalla Pozza R (2015). Intima media thickness measurement in children: A statement from the Association for European Paediatric Cardiology (AEPC) Working Group on Cardiovascular Prevention endorsed by the Association for European Paediatric Cardiology. Atherosclerosis.

[42] Geelhoed JJ (2009). Reliability of renal ultrasound measurements in children. Pediatric nephrology.

[43] de la Puente ML, Canela J, Alvarez J, Salleras L, Vicens-Calvet E (1997). Cross-sectional growth study of the child and adolescent population of Catalonia (Spain). Annals of human biology.

[44] Haenggi MH, Pelet J, Guignard JP (1999). Estimation of glomerular filtration rate by the formula GFR = K × T/Pc. Archives de pediatrie: organe officiel de la Societe francaise de pediatrie.

[45] Dinkel E (1985). Kidney size in childhood. Sonographical growth charts for kidney length and volume. Pediatric radiology.

